# Assessment of Driver’s Drowsiness Based on Fractal Dimensional Analysis of Sitting and Back Pressure Measurements

**DOI:** 10.3389/fpsyg.2018.02362

**Published:** 2018-11-29

**Authors:** Atsuo Murata, Ippei Kita, Waldemar Karwowski

**Affiliations:** ^1^Department of Intelligent Mechanical Systems, Okayama University, Okayama, Japan; ^2^Department of Industrial Engineering and Management Systems, University of Central Florida, Orlando, FL, United States

**Keywords:** drowsiness, crash, back pressure, sitting pressure, fractal dimension, nonlinear dynamics, self-similarity, unpredictability

## Abstract

The most effective way of preventing motor vehicle accidents caused by drowsy driving is through a better understanding of drowsiness itself. Prior research on the detection of symptoms of drowsy driving has offered insights on providing drivers with advance warning of an elevated risk of crash. The present study measured back and sitting pressures during a simulated driving task under both high and low arousal conditions. Fluctuation of time series of center of pressure (COP) movement of back and sitting pressure was observed to possess a fractal property. The fractal dimensions were calculated to compare the high and low arousal conditions. The results showed that under low arousal (the drowsiness state) the fractal dimension was significantly lower than what was calculated with high arousal. Accumulated drowsiness thus contributed to the loss of self-similarity and unpredictability of time series of back and sitting pressure measurement. Drowsiness further reduces the complexity of the posture control system as viewed from back and sitting pressure. Thus, fractal dimension is a necessary and sufficient condition of a decreased arousal level. It further is a necessary condition for detecting the interval or point in time with high risk of crash.

## Introduction

Three types of measures are used to assess driver drowsiness: Bio-signal-based, vehicle-based and behavioral measures. Bio-signal-based measures include electroencephalography (EEG), heart rate variability (HRV) and certain ocular measures, such as pupil diameter, blink frequency and percentage eye closure (PERCLOS) (Skipper and Wierwillie, 1986; Brookhuis and Waard, 1993; Galley, 1993; Wright and McGown, 2001; Hanowski et al., 2008; Murata and Hiramatsu, 2008; Murata and Nishijima, 2008; Chen and Jin, 2012). Among vehicle-based measures are lane position, line crossing and steering wheel inputs. Back, foot and sitting pressure are classified as behavioral measures (Murata et al., 2013; Murata, 2016; Murata and Fukuda, 2016). Behavioral and vehicle-based measures possess the practical advantage of noninvasive measurement relative to physiological measurement.

Prior studies have found a correlation between driver-based measures and subjective ratings of drowsiness. Kurian and Rishikesh (2013) examined changes in electrocardiography (ECG), EEG and eye closure with increasing drowsiness. Mehler et al. (2012) attempted to differentiate multiple levels of cognitive work demand changes using heart rate and skin conductance. Both measures were higher as cognitive demand increased. Reimer et al. (2012) applied eye-gaze concentration to assess a driver’s cognitive workload. Their study demonstrated greater eye-gaze concentration as task difficulty increased. Reimer and Mehler (2011) used heart rate and skin conductance levels to characterize cognitive workload during actual highway driving. In so doing they observed a consistent pattern of change in heart rate with a higher measure of cognitive workload. Murata (2018) applied Bayesian theory to EEG, heart rate variability (HRV) and tracking error during a simulated driving task. A proposed method of evaluating drowsiness revealed increased posterior probability of drowsiness states toward the end of the experiment.

Other studies have assessed, classified and predicted drowsiness states (Ji et al., 2004, 2006; Sayed et al., 2012; Singh and Banga, 2013; Kusuma and Sunitha, 2014; Samiee et al., 2014). However, no definite or effective methods emerged from this research for determining when a warning signal should be presented to the driver. The psychological rating of drowsiness may be predicted using behavioral and physiological measures (Murata et al., 2013; Samiee et al., 2014). However, these methods cannot automatically detect the point in time when high risk of crash is triggered using a criteria that is based on the decreased arousal level from a variety of bio-signals and behavioral-signals that is sensitive to drowsiness.

Murata (2016), Murata and Fukuda (2016), Murata et al. (2017), and Murata (2018) employed an *X*-bar control chart or Bayesian theory in a simulated driving task. In doing so they succeeded to some extent in identifying the point in time of high risk of crash before a virtual crash actually occurred. However, traditional time series analysis techniques such as an *X*-bar control chart and a renewal of Bayesian posterior probability have limitations. For one, they do not consider the nonlinear dynamics of a complicated biological system. They also fail to provide a perfect model for identifying the state of high risk of crash.

The approaches taken in Murata (2016), Murata and Fukuda (2016), Murata et al. (2017), and Murata (2018) require careful and continuing tracking of changes to identify the high risk point in time. This aspect of these studies makes it more difficult to assess accumulated drowsiness. The studies also do not reflect drowsiness induced over a long period of time, nor can they globally and simply represent the current state. Lastly, they do not account for variability in the assessment indices or decrease in the complexity of the posture control system. The variability of different behavioral measures makes it difficult to assess drowsiness and thereby determine the state of high risk of crash. However, this variability may be expressed appropriately using a single measure.

The process of falling asleep has been extensively characterized by stages of sleep (I-IV) (Andreassi, 1980; Grandjean, 1990). However, its complexity makes it difficult to predict with complete certainty the onset of drowsy driving. Because sleep-awakening regulation is among the more complicated biological systems, it must be directed by nonlinear dynamics of unpredictability and self-similarity. Until now, nonlinear dynamics of behavioral measures such as sitting and back pressure accompanied by accumulated drowsiness had not been explored. Yet they may provide the insights necessary for identifying a state of high risk of crash. If used with existing detection techniques, nonlinear dynamics that change over long time intervals may enhance the detection technique of point in time or interval with high risk of crash.

Since its introduction, fractal dimension has been used to investigate nonlinear dynamics of a variety of biological and medical phenomena. In particular, fractal dimension was used to quantify the complexity of dynamic fluctuation of biological systems. It is well known that nonlinear chaotic dynamics are ubiquitous in many biological systems, such as EEG, pulsation in capillary vessels, body sway and HRV (Murata and Iwase, 1998, 2001; Iwase and Murata, 2002, 2004). Rapp et al. (1989), Arle and Simon (1990), Glenny et al. (1991), Lutzenberger et al. (1992, 1995), and Murata and Iwase (2001) used fractal dimension to evaluate cerebral brain activity associated with changes in cognitive workload. These studies showed that the unpredictability and self-similarity of the time series of EEG activities increased with higher mental workload. When mental workload is higher, EEG activities are directed by a more complex mechanism. Fractal dimension is one of these mechanisms. Although a mental task was not used, Liu et al. (2005) identified the relationship between fractal dimension of EEG and %MVC (Maximum Voluntary Contraction) (handgrip force). These findings suggested that fractal dimension is part of the central nervous system that controls activities such as mental arithmetic or manual lifting. Fractal dimension is effective in extracting the information necessary to explore the central nervous system functions that direct certain mental and physical activities. Therefore, fractal dimensions should increase when the brain works more actively except for slept state (during not stages of sleep but arousal or decreased arousal state).

Fractal dimension (Mandelbrot and Hudson, 2004) and self-similarity measure and quantify the complex dynamic fluctuations in time series signals (West, 2013). The complexity found in chaos is different. Chaos-related complexity is determined by a simple equation with only a few dynamic variables. Chaos, therefore, can be predicted to some extent over short time intervals. It also can express randomness, including irregularity and disorder, but only in part. Randomness, in turn, may be expressed by an infinite number of elements, though only in an unknown way, not a deterministic one. Fractal dimension more appropriately reflects the complexity caused by randomness of time series. Randomness caused by a dynamic system is uncontrollable and unpredictable. Ordinarily, a dynamic system having an infinite number of unknown elements disrupts the system in a random and unknown way. This is what makes the system’s behavior unpredictable and uncontrollable. But a dynamic system with a few elements exhibits a deterministic and simple behavior. This system provides limited predictability and controllability peculiar to chaos. It is possible that fractal dimension alone can be used to assess the complexity of the time series data of COP movement of back or sitting pressure. Moreover, there are few studies that attempted to assess drowsiness using a nonlinear analysis method of postural control system accompanied by increased drowsiness.

This study hypothesized that nonlinear dynamics, specifically fractal properties, can be observed in the back and from sitting pressure while driving. More specifically, different fractal properties of the back and of sitting pressure are detected between the low and high arousal conditions. Understanding fractal dimensions that change over a long period of time may shed light on the detection of drowsiness while driving.

## Materials and Methods

### Participants

Nineteen healthy male undergraduate students, ages 21–24, took part in the experiment. The visual acuity of all participants exceeded 20/20. None reported any orthopedic or neurological diseases over the last 3 years. Each participant signed an informed consent for their participation in the experiment.

Participants were selected after it was confirmed that they had received no medical treatment, nor experienced any serious medical conditions over the previous 3 years. They also were required not to be on a medically-necessary food diet. The experiment was checked on standard bio-security and institutional safety procedures and approved by the Ethical Committee of the Department of Intelligent Mechanical Systems, Okayama University.

### Apparatus

**FIGURE 1:**
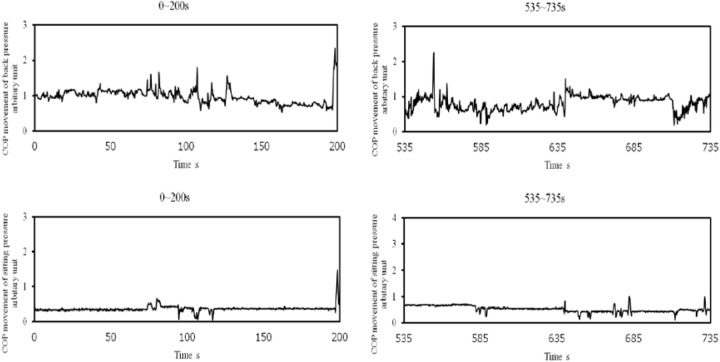
Samples of COP movement of back (upper) and sitting (lower) pressure during 200 s from the start and during 200 s before the virtual crash (Participant B).

The experimental system consisted of a display of a simulated driving task and a steering wheel used for the task. The detailed display of the inside lane was the same as that used by Murata et al. (2013, 2017). It consisted of three lanes, each of which was 3.6 m wide. Measurement systems were common to both sitting and back pressure. Two pressure measurement sheets (Nitta, SR Soft Vision), each 450 mm × 450 mm, were attached to both the sitting surface and the back surface of a seat to measure sitting and back pressure, respectively. The same apparatus was used to measure the center of pressure (COP) with a sampling frequency of 5 Hz (The measurement system sampled COP every 0.2 s). The movement of COP (movement from *n*-th COP to (*n*+1)-th COP) was calculated every 0.2 s for both back and sitting pressure. Examples of COP movement for both back and sitting pressure are depicted in Figure 1. The larger values in the figure show that great body movement occurred. Time series of COP movement was used for the fractal dimensional analysis, because chaotic property of COP fluctuations was pointed out in Murata and Iwase (1998).

### Task

Each participant sat on an automobile seat to perform the simulated driving task (For more detail, refer to Murata et al., 2013, 2017). The task required participants to follow a preceding car while maintaining a moderate distance between the preceding car and their own car. So long as the participant held the moderate distance between the two cars, the preceding car would be surrounded by a green rectangle. However, if the distance between the two vehicles became too short or too long, the color of the rectangle would change to a different color (red if the distance became too short, or blue if the distance grew too long). Participants also were required to steer their vehicle with the steering wheel and keep the center of their vehicle to a center line (displayed in purple color in the actual experiment) as much as possible.

### Procedure

Participants appeared at the laboratory at 8:00 p.m. They were required to remain awake there the entire night. The experiment began at 6:00 a.m. During the sleep deprivation, no one was permitted to consume caffeinated drinks or exercise excessively. In addition, screen time – such as interacting with a personal computer or a smart phone – was limited to less than an hour. The sleep-wake history of participants was controlled so that they spent their usual life of sleeping and eating, although actigraph was not used. The reported sleep duration of participants ranged from 7 to 9 h (mean: 7.34 h and standard deviation: 0.65 h). They also were required to finish their dinner until they appear at the laboratory, and were not allowed to eat until the experiment was over. These conditions were intended to induce and promote drowsiness or a low state of arousal during the experiment.

Back pressure was used because it has been observed that drowsy drivers tend to move forward or backward according to their accumulated drowsiness. Forward movements produce less pressure on the backrest of a driver’s seat. Backward movements exert greater pressure. Although it has been suggested that there are differences of COP movement of back and sitting pressure between high and low (sleep-deprived) state (Murata et al., 2013), it also is pointed out that it is difficult to differentiate the degree of drowsiness using such measures. As demonstrated in Figure 1, it is difficult to detect the difference of COP movement between the first and the second half of measurement of COP movement. Therefore, the motivation of this study was to further analyze time series of COP movement of back and sitting pressure using fractal dimensional analysis to get further insights into how nonlinear dynamics of postural control system changes with the change in degree of drowsiness.

The behavioral measures discussed above were recorded while participants performed the simulated driving task under a low arousal condition for no more than 60 min. When 60 min had passed, the experimenter terminated the experiment. As the experiment progressed, the experimenter observed the degree of drowsiness and the declined behavior. Symptoms of these conditions included extreme tracking error, suspension of steering operation and the like. When the experimenter judged that the participant was nearly falling asleep and unable to continue the task any further, the experimenter decided on whether or not to continue the experiment. This decision was made in accordance with the criterion discussed below for identifying the point in time of a virtual crash.

The degree of drowsiness varied among the participants. As a result, the process of drowsiness development and the duration of the experimental task varied as well. Moreover, for 15 out of the 19 participants, the experiment terminated before the 60 min period had passed.

The same procedure was followed, and data recorded, on a separate day. This second experimental task was carried out under high arousal and without sleep deprivation. The duration limit for this task was 20 min.

The point in time of virtual crash was identified on the basis of the tracking error in the main driving task and the observation of behavior of participant according to the criteria in Murata et al. (2017). Two experimenters monitored the participants’ state to ensure objectivity and consistency in the judgment process. Both applied two conditions to determine whether a participant would have encountered a crash with certainty in the real world if he or she had continued driving. In that event the corresponding point in time was identified as that of the virtual crash. The two triggering conditions were: (i) The participant had dozed off for more than 1 min, and (ii) The mean tracking error per minute was sustained at more than 1.8 m (half of the lane width) for 30 s. The implication of the second condition was that the virtual vehicle had substantially deviated from the lane and could not be judged to be driving normally. If condition (i) was satisfied, condition (ii) was checked.

Throughout the experiment, the two experimenters observed the participant using consistent criteria to confirm whether or not condition (i) was satisfied. The criteria included change in posture, such as bending forward or backward tilting; steering wheel movement; and eye closure. The point in time of virtual crash was certified only when the two experimenters’ judgments agreed on conditions (i) and (ii). The participant also was required to agree with the experimenters’ judgment after the experiment was over. The reason why physiological measures were not used to identify the point in time of virtual crash is as follows. The attachment of EEG measurement cap is troublesome for participants and it is possible enough to identify the point in time of virtual crash on the basis of the deviation of participant’s vehicle from the center of the simulated lane and the behavioral observation of participants by two experimenters.

The fractal dimension of time series of COP movement of back and sitting pressure was calculated according to the method described in the next section. The calculation was made for a 1200 s-high arousal period and for the duration of the sleep deprivation experiment. That meant calculating from the start of the experiment to the point in time of virtual crash or the end of the experiment. The duration differed among participants. The fractal dimension also was calculated 200 s from the start of the experiment and for 200 s before the virtual crash or end of the experiment.

## Fractal Dimension

**FIGURE 2:**
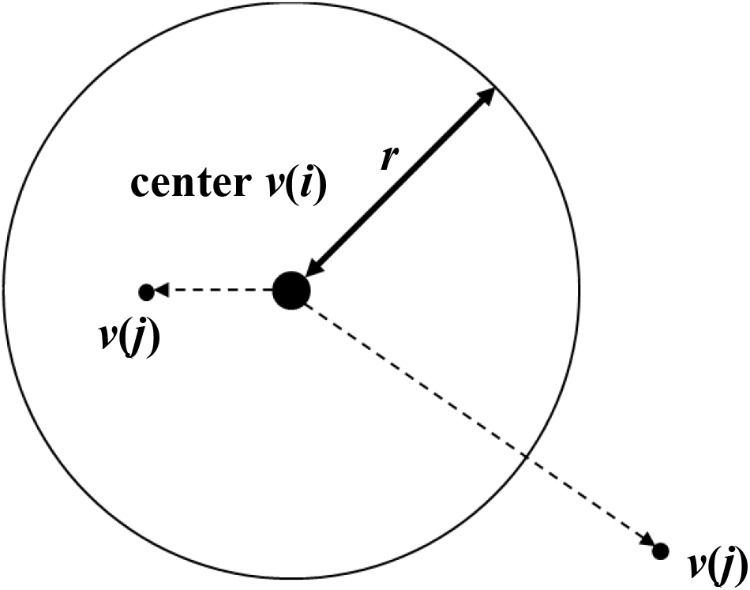
Calculation procedure of correlation integral.

The GP (Grassberger – Procaccia) method (Grassberger and Procaccia, 1983a,b) was used to calculate fractal dimension of the time series of back and sitting pressure. The correlation integral defined by Equation (1) was calculated according to the procedure below:

(1) $$C_{m}(r)=\underset{N\to \infty }{\lim}\frac{1}{N^{2}}\sum_{\begin{array}{l} i,j=1\\ \;\;\;\;i\neq j \end{array}}^{N}H(r-|v(i)-v(j)|))$$

where *H* is the Heaviside function, and *r* is the radius of the *m*-dimensional sphere, the center of which corresponds to *v*(*i*) (see Figure 2). The calculation was checked, and then counted whether (*N*-1) points *v*(*j*) (*j* = 1,2,…..,*N, i*≠*j*) other than *v*(*i*) is within the sphere. Ulimately, the correlation integral in Equation (1) was calculated. If the correlation integral was expressed by the following equation, then the correlation dimension equaled *v*(*m*):

(2) $$C_{m}(r)\propto r^{v(m)}$$

(3) $$\log_{10}C_{m}(r)=\log_{10}k_{0}+v(m)\;\log_{10}r$$

**FIGURE 3:**
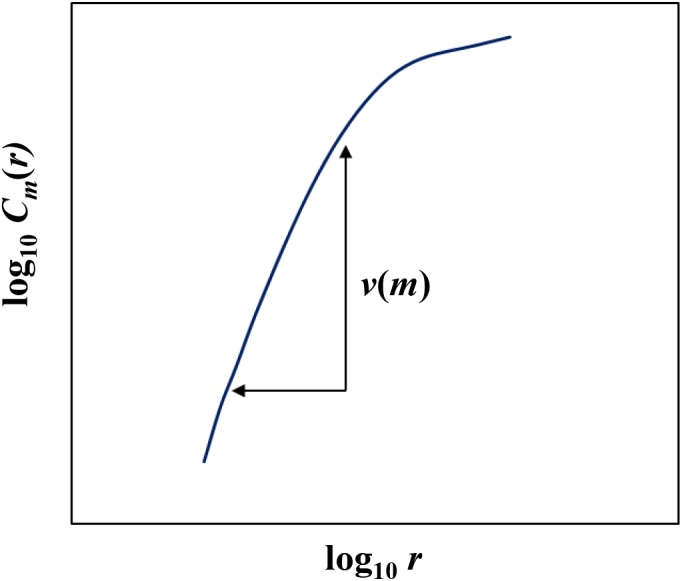
Calculation of correlation dimension.

If Equation (3) held, then self-similarity held. In that case the correlation dimension *v*(*m*) could be calculated as shown in Figure 3. Because the genuine correlation dimension is unknown, the correlation dimension *v*(*m*) was calculated as a function of *m*. If the time series of the system had self-similarity property, *v*(*m*) saturated with the increase in *m*. This corresponded to fractal dimension *d*. Fractal dimension *d* must satisfy the following inequality according to Ruelle (1990):

(4) $$d\leq \log_{10}N^{2}$$

The value of time lagτ was empirically determined as 0.6 s.

## Results

**FIGURE 4:**
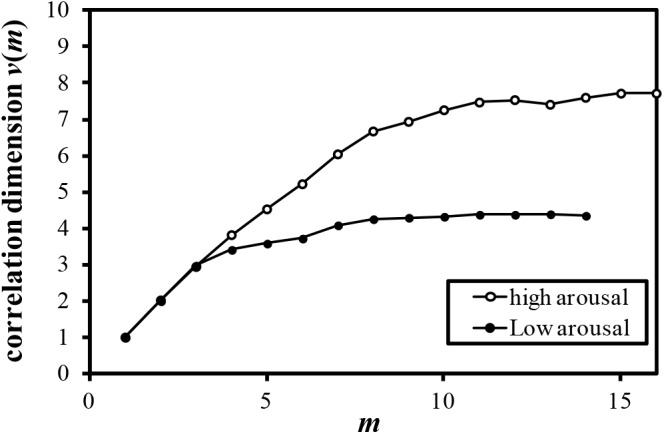
Relationship between *m* and correlation dimension *v*(*m*) for COP movement of back pressure (Participant K).

**FIGURE 5:**
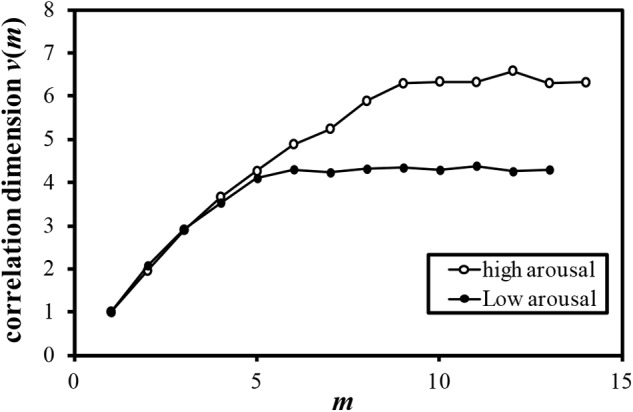
Relationship between *m* and correlation dimension *v*(*m*) for COP movement of back pressure (Participant L).

**FIGURE 6:**
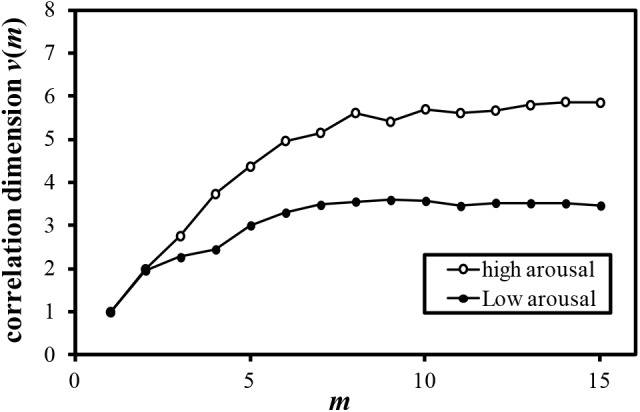
Relationship between *m* and correlation dimension *v*(*m*) for COP movement of sitting pressure (Participant M).

**FIGURE 7:**
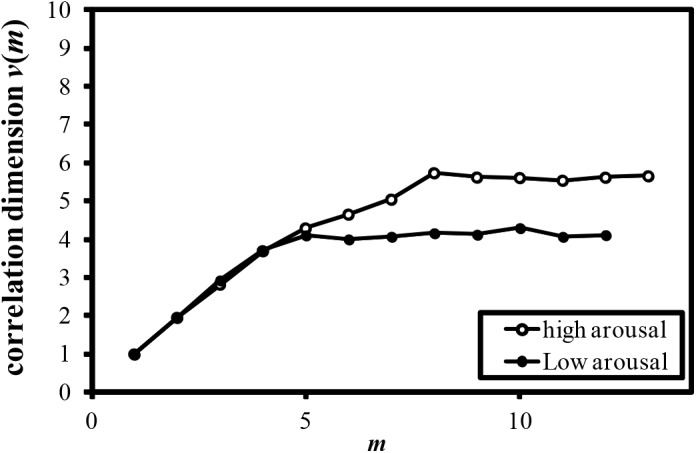
Relationship between *m* and correlation dimension *v*(*m*) for COP movement of sitting pressure (Participant N).

Examples of the relationship between the dimension of phase space *m* and the correlation dimension *v*(*m*) are plotted in Figures 4–7. Figures 4–7 correspond to the fractal dimension of COP movement of back and sitting pressure, respectively. Fractal dimension was obtained for both back and sitting pressure among all nineteen participants. As shown in the previous section, *v*(*m*) saturated with the increase in *m* and satisfied the condition of Equation (4). Fractal dimension was calculated as a mean of saturated 5–6 correlation dimensions at higher values of *m* as shown in Figures 4–7.

The points in time of virtual crash identified in the experiment are summarized in Table 1. Table 1 also shows the comparison of fractal dimension for COP movement of both back and sitting pressure between high and low (sleep deprivation) arousal between 200 s after the start of the experiment and 200 s before the point in time of virtual crash or end of the experiment. For all of 19 participants, fractal dimension of back and sitting pressure under the high arousal state was higher than that under the low arousal (sleep-deprivation) state. The duration of measurement under the low arousal state differed among participants. The fractal dimensions also were calculated for the low and high arousal states during the 20 min task.

**Table 1:** Results of identification of point in time of virtual crash.

Participant	Point of time of virtual crash	FD(High arousal) vs. FD(Low arousal)	FD(200 s from start) vs. FD(200 s before vc or end)
A	No	FD(High arousal) > FD(Low arousal)	FD(200 s from start) > FD(200 s before end)
B	735 s	FD(High arousal) > FD(Low arousal)	FD(200 s from start) > FD(200 s before vc)
C	775 s	FD(High arousal) > FD(Low arousal)	FD(200 s from start) > FD(200 s before vc)
D	1290 s	FD(High arousal) > FD(Low arousal)	FD(200 s from start) > FD(200 s before vc)
E	807 s	FD(High arousal) > FD(Low arousal)	FD(200 s from start) > FD(200 s before vc)
F	No	FD(High arousal) > FD(Low arousal)	FD(200 s from start) > FD(200 s before end)
G	817 s	FD(High arousal) > FD(Low arousal)	FD(200 s from start) > FD(200 s before vc)
H	1987 s	FD(High arousal) > FD(Low arousal)	FD(200 s from start) > FD(200 s before vc)
I	728 s	FD(High arousal) > FD(Low arousal)	FD(200 s from start) > FD(200 s before vc)
J	1078 s	FD(High arousal) > FD(Low arousal)	FD(200 s from start) > FD(200 s before vc)
K	1222 s	FD(High arousal) > FD(Low arousal)	FD(200 s from start) > FD(200 s before vc)
L	629 s	FD(High arousal) > FD(Low arousal)	FD(200 s from start) > FD(200 s before vc)
M	978 s	FD(High arousal) > FD(Low arousal)	FD(200 s from start) > FD(200 s before vc)
N	2076 s	FD(High arousal) > FD(Low arousal)	FD(200 s from start) > FD(200 s before vc)
O	No	FD(High arousal) > FD(Low arousal)	FD(200 s from start) > FD(200 s before end)
P	525 s	FD(High arousal) > FD(Low arousal)	FD(200 s from start) > FD(200 s before vc)
Q	No	FD(High arousal) > FD(Low arousal)	FD(200 s from start) > FD(200 s before end)
R	719 s	FD(High arousal) > FD(Low arousal)	FD(200 s from start) > FD(200 s before vc)
S	312 s	FD(High arousal) > FD(Low arousal)	FD(200 s from start) > FD(200 s before vc)


*FD, fractal dimension (back and sitting pressure); vc, virtual crash.*

**FIGURE 8:**
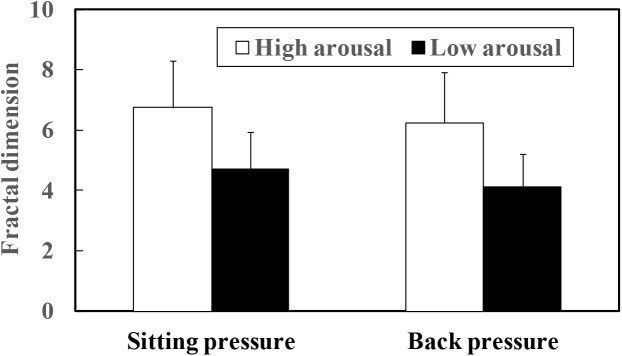
Fractal dimension of COP movement of sitting and back pressure compared between high arousal and low arousal (drowsy) state.

**FIGURE 9:**
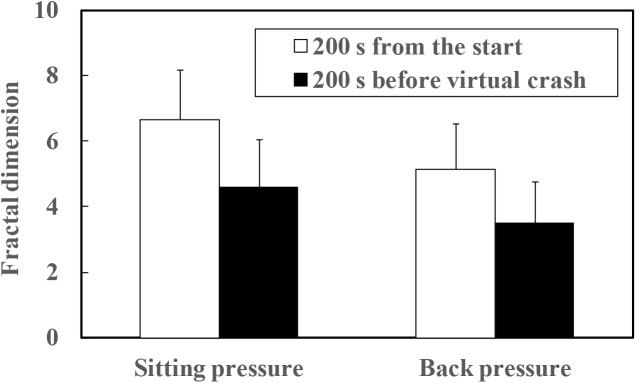
Fractal dimension of COP movement of sitting and back pressure compared between high arousal and low arousal (drowsy) state.

The mean fractal dimensions of both sitting and back pressure are plotted as a function of arousal level in Figure 8. The fractal dimensions of four participants (A, F, O, and Q in Table 1) for whom the point in time of virtual crash was not detected also were included in the data set out in Figure 8. As a result of the paired *t*-test, significant differences were detected between the high and low arousal states for both sitting (*t* = 8.077, *p* < 0.01) and back pressure (*t* = 6.441, *p* < 0.01). Fractal dimension was higher during the high arousal state than during low arousal. Fractal dimension also was calculated during the sleep deprivation experiment. That calculation included the data gathered for the periods 200 s after the start of measurements and 200 s before the virtual crash or end of the experiment. The results are plotted in Figure 9. As for the four participants with a virtual crash not detected, the data for 200 s immediately before the end of the experiment were used to calculate fractal dimension. A paired *t*-test revealed significant differences between the 200 s duration after the start of measurements and the 200 s duration before the virtual crash or the end of the experiment for both sitting (*t* = 7.357, *p* < 0.01) and back pressure (*t* = 4.105, *p* < 0.05). The fractal dimension during 200 s immediately after the start of measurements was significantly larger than it was during 200 s before the virtual crash or the end of the experiment.

## Discussion

The fluctuation of time series of COP movement of back and sitting pressure showed a fractal property represented by self-similarity and unpredictability, because the correlation dimension saturated at higher values of m as shown in Figures 3–6. The fractal dimension under low arousal (drowsiness state) was significantly less than that found under high arousal (see Figures 8, 9). This strongly suggests that accumulated drowsiness eliminates self-similarity and unpredictability of time series of back and sitting pressure. Moreover, the complexity of the posture control system decreased when the subject was drowsy.

As shown in Table 1, the point in time of virtual crash was not identified for four participants (Participant A, F, O, and Q). Application of an algorithm (Murata et al., 2017) also prevented detection of the point in time with high risk of crash for these four participants. These findings do not necessarily mean that the four participants maintained a high arousal level during the measurement. For all participants, fractal dimension of COP movement of back and sitting pressure under the high arousal state was higher than that under the low arousal (sleep-deprivation) state (see Figures 8, 9). In fact, their arousal levels decreased to some extent. These participants simply did not encounter a virtual crash during the simulated driving task. As shown in Figure 9, the fractal dimension for these four participants tended to be significantly lower before the experiment ended. This suggests that fractal dimension can be a precursor of a forthcoming high risk of crash, and can be used to detect decreased arousal level even if the point in time of virtual crash is not detected. As shown in Figure 1, simply measuring COP movement does not enable us to detect the decreased state of arousal level. Thus, as hypothesized, it might be concluded that the fractal dimensional analysis of COP movement of back and sitting pressure is promising for the detection of decreased arousal level. This would be true even for the four participants who did not encounter a virtual crash during the measurement.

The present study suggests that decreasing fractal dimension may be a necessary and sufficient condition of a state of drowsiness. Thus, if a driver is feeling drowsy, the fractal dimension decreases. Conversely, observing a decrease in fractal dimension suggests that the driver is experiencing some degree of drowsiness. Decreasing fractal dimension also may be a necessary condition for detecting the point in time of virtual crash or high risk of virtual crash. However, decreasing fractal dimension does not necessarily lead to an actual virtual crash; nor is it a sufficient condition of a virtual crash or high risk of virtual crash. If a virtual crash occurs or a high risk of virtual crash is detected, then fractal dimension decreases. Fractal dimension also decreases as arousal level decreases.

The tendency of fractal dimension to change correspondingly to arousal level was consistent regardless of the duration of analysis interval. As shown in Figures 8, 9, similar tendencies (decrease of fractal dimension under low arousal and drowsiness states) were observed for the 20 min high arousal and low arousal intervals. The same findings were made for the interval between 200 s after measurements were started and 200 s before the virtual crash. Decreased fractal dimension also was consistently observed during the 200 s immediately before the virtual crash and the end of the experiment. Table 1 further validates these results. These results are indicative of the decreased complexity of the postural control system under a low arousal state. Although the decrease of fractal dimension is a necessary condition, it does not necessarily induce a virtual crash or drowsiness state with a high risk of crash. It may be that the decrease is attributable to drowsiness and reflects to some extent the risk of inducing a low arousal state leading to a crash.

Many studies have verified that fractal dimension increases when the brain works actively to compensate for high mental or physical workload (Rapp et al., 1989; Lutzenberger et al., 1992, 1995; Murata and Iwase, 2001; Liu et al., 2005). It therefore follows that drowsiness occurs when the brain is not working actively, thus further demonstrating this study’s consistency with past findings.

As discussed earlier, fractal dimension (self-similarity and unpredictability) decreased for four of the participants even without a detection of virtual crash. However, detecting at least the appearance of drowsiness still may be a first step toward detecting the interval or point in time with high risk of crash. If fractal dimension significantly decreases, we proceed to the second step for detecting the interval or point in time with high risk of crash using the method by Murata (2016), Murata and Fukuda (2016), Murata et al. (2017), and Murata (2018).

The results further verified the hypothesis that nonlinear dynamics in the time series of back and sitting pressure have a fractal property. This property is weakened while driving under a low arousal state. In particular, different nonlinear dynamics of back and sitting pressure were observed between the low and the high arousal conditions. The low fractal dimension consistently manifested itself for time series of both back and sitting pressure. Fractal dimension’s changes over long time intervals may shed light on the detection of drowsy driving. It also may enhance our ability to identify a state with high risk of crash if used together with the techniques already developed by Murata (2016), Murata and Fukuda (2016), Murata et al. (2017), and Murata (2018). Furthermore, a detection of a decrease in fractal dimension over a fixed interval may reveal the point in time or interval with high risk of crash using the methods proposed by Murata (2016), Murata and Fukuda (2016), Murata et al. (2017), and Murata (2018). The overall result will be increased reliability of detection.

One limitation of this study was the calculation of fractal dimension using data for at least 200 s. Using a shorter period, possibly 30 s, would allow both the first step of checking for drowsiness and the second step of detecting the point in time of interval with high risk of crash to proceed with greater frequency. Checking with greater frequency also would enhance the detection accuracy of the point in time or interval with high risk of crash.

Future research should combine fractal dimensional analysis of sitting and back pressure with the detection method for point in time with high risk of crash. This also should improve the reliability of the detection method. Future research also should increase the number of participants and include a variety of samples other than 21–24 male participants.

## Conclusion

This study measured sitting and back pressures in a simulated driving task under high and low arousal conditions. It further attempted to assess drowsiness on the basis of fractal dimensional analysis. The results suggested that fluctuations of time series of COP movement of back and sitting pressure have a fractal property. The fractal dimension is significantly lower under a low arousal or drowsy state than it is under high arousal. Thus, accumulated drowsiness eliminates self-similarity of time series of back and sitting pressure. Drowsiness also reduces the complexity of the posture control system. Finally, decreased fractal dimension is a necessary and sufficient condition of a decreased arousal level. It further is a necessary condition for detecting the interval or point in time with high risk of crash.

## Author Contributions

AM formed a hypothesis and designed this study, planned and carried out an experiment, analyzed the data and discussed this with co-authors. IK conducted an experiment, and calculated fractal dimension, and discussed the results with co-authors. WK checked the validity of research hypothesis and research plan with co-authors, and discussed the data from the viewpoints of safety management. All authors wrote the manuscript and jointly approved the final manuscript for submission.

## Conflict of Interest Statement

The authors declare that the research was conducted in the absence of any commercial or financial relationships that could be construed as a potential conflict of interest.
